# Correlation between myocardial radiomics features at the left atrium–left atrial appendage junction and stroke risk in atrial fibrillation patients: a retrospective case–control study

**DOI:** 10.3389/fcvm.2026.1817530

**Published:** 2026-09-23

**Authors:** Xiao-xuan Wei, Xin Tian

**Affiliations:** Department of Medical Imaging, The Second Hospital of Hebei Medical University, Shijiazhuang, China

**Keywords:** atrial fibrillation (AF), cardiac CT, myocardium tissue, radiomics, stroke

## Abstract

**Objective:**

This study aimed to systematically evaluate the microstructure changes of the myocardium at the left atrium–left atrial appendage (LA–LAA) junction in patients with atrial fibrillation (AF)-related cardioembolic stroke, and to explore its potential role as an imaging marker associated with stroke.

**Methods:**

This retrospective study included four groups: AF-related cardioembolic stroke group (*n* = 60), AF-non-stroke group (*n* = 29), non-AF-stroke (*n* = 53), and non-AF-non-stroke (*n* = 53). All subjects underwent cardiac computed tomography examination. Myocardial thickness at the LA–LAA junction was measured, and radiomics features were extracted. Radiomics features included first-order statistical features, shape features, and texture features. Least Absolute Shrinkage and Selection Operator (LASSO) regression was used to screen features, and a logistic regression model combining radiomics features and clinical parameters was constructed. Group comparisons were performed using t-tests or Mann–Whitney U tests. The predictive ability of the model was evaluated with the receiver operating characteristic (ROC) curve, and a nomogram was used for risk assessment.

**Results:**

Compared with AF patients without stroke, patients with AF-related cardioembolic stroke significantly increased myocardial thickness at the LA–LAA junction [7.1 (6.43–8.33) mm vs. 6.58 ± 1.45 mm, *p* < 0.05]. In addition, multivariate logistic regression demonstrated that myocardial thickness was independently associated with AF-related cardioembolic stroke. Three radiomics features were selected by LASSO regression in AF-related cardioembolic stroke and non-AF-non-stroke patients, and a radiomics score (Rad-score) was generated. The Rad-score was statistically significant between the groups (*p* < 0.001). The combined model incorporating Rad-score and clinical parameters achieved an area under curve (AUC) of 0.968 in the test cohort.

**Conclusion:**

Both myocardial radiomics features and myocardial thickness at the LA–LAA junction may serve as potential imaging markers associated with AF-related cardioembolic stroke. Their incremental value for stroke risk stratification beyond established clinical risk factors requires further validation in larger prospective cohorts.

## 1 Introduction

Atrial fibrillation (AF) is one of the most common cardiac arrhythmias (1), associated with severe complications, including stroke, thromboembolism, and cognitive impairment (2). AF increases the risk of ischemic stroke three- to fivefold (3, 4). AF-related cardioembolic stroke accounts for about 25%–30% of all ischemic strokes, with greater disability and higher mortality rates (5). Clinically, the CHA_2_DS_2_-VASc score is used to assess the risk of stroke in AF patients, but patients with low CHA_2_DS_2_-VASc score may still experience stroke and thromboembolic events. Other imaging markers should be considered to predict the risk of stroke more precisely.

Imaging assessment of the left atrium (LA) and left atrial appendage (LAA) may provide complementary information to the CHA_2_DS_2_-VASc score for thromboembolic risk stratification in patients with AF. The LAA is the predominant site of thrombus formation in AF, and impaired LAA emptying promotes blood stasis and thrombus formation. AF-related myocardial fibrosis may contribute to impaired LAA mechanical function (6, 7). Reduction of emptying function increases blood flow stasis in the chamber of LAA and creates a prothrombotic milieu, which promotes thrombus formation (8). Once the thrombus is dislodged, it can be embolized into the cerebral circulation with arterial blood flow, causing focal cerebral ischemia and hypoxia, ultimately leading to cardioembolic ischemic stroke (9). Cardiovascular magnetic resonance (CMR) imaging is widely used to detect myocardial fibrosis (10, 11). However, assessing myocardial fibrosis in the LA and LAA is challenging due to the thin myocardium, which limits the accuracy of conventional imaging techniques in evaluating myocardial remodeling.

Radiomics, a rapidly evolving analytical method, offers a novel approach to quantitatively evaluate myocardium. By extracting and analyzing a large number of image features, radiomics can identify patterns and biomarkers that support diagnosis, prognosis, and therapeutic decision-making (12). The process includes image acquisition and preprocessing, region-of-interest (ROI) delineation, radiomics feature extraction, feature dimensionality reduction, and model establishment (13). Radiomics features are quantitative imaging markers extracted from medical images, which can capture subtle variations in tissue characteristics that may not be visually detectable. These features are generally classified into three categories: first-order statistical features, shape features, and texture features. First-order features describe the distribution characteristics of voxel intensity values within the ROI, including mean, median, variance, entropy, kurtosis, and skewness. Shape features characterize the geometric properties of the ROI, such as volume, surface area, sphericity, and compactness. Texture features quantify spatial heterogeneity and relationships among voxel intensity values, including features derived from gray-level co-occurrence matrix (GLCM), gray-level run-length matrix (GLRLM), gray-level size zone matrix (GLSZM), gray-level dependence matrix (GLDM), and neighboring gray-tone difference matrix (NGTDM) (14). These features of the myocardium may reflect microscopic myocardial heterogeneity related to atrial fibrosis, extracellular matrix remodeling, and local structural alterations. Radiomics has been extensively applied in cardiovascular and cerebrovascular disease research (15, 16). For example, myocardial radiomics using CMR can predict cardiovascular events (17), while cardiac CT radiomics has demonstrated potential in predicting AF (1). Despite these advancements, research on the association between myocardium at the LA–LAA junction and the risk of AF-related cardioembolic stroke is relatively limited. This study therefore aimed to systematically evaluate the microstructure changes of myocardium at the LA–LAA junction in patients with AF-related cardioembolic stroke, and to explore its potential role as an imaging marker associated with stroke.

## 2 Methods

### 2.1 Research object

A retrospective analysis was conducted on 89 AF patients and 106 patients without AF who underwent cardiac CT examination at our hospital between January 2021 and November 2024.

The AF group included patients from the AF-related cardioembolic stroke group and AF-non-stroke group, whereas the non-AF group included patients from the non-AF-stroke group and the non-AF-non-stroke group. Relevant clinical information was recorded for all participants. The inclusion criteria for the AF group were as follows: (1) AF was confirmed by physical examination and electrocardiogram examination in patients; (2) 256-slice spiral cardiac CT examination was performed in all cases. The inclusion criteria for the non-AF group were as follows: (1) no clinical history or electrocardiographic evidence of AF; (2) no evidence of coronary artery disease or structural cardiac abnormalities on 256-slice cardiac CT; and (3) patients with other comorbidities, such as hypertension or diabetes mellitus, were not excluded.

The exclusion criteria for both the AF group and the non-AF group were as follows: (1) poor-quality cardiac CT images that made it difficult to measure myocardial thickness; (2) patients with implanted cardiac pacemakers and other implants; and (3) patients with myocardial infarction, hypertrophic cardiomyopathy, and other heart diseases related to myocardium, arrhythmias, coronary heart disease, cerebral hemorrhage, and arteriosclerosis.

The inclusion criteria for the AF-related cardioembolic stroke group required the presence of AF and ischemic stroke with imaging features suggestive of cardioembolism. Cardioembolic stroke was determined based on a comprehensive evaluation of clinical information, cardiac assessment, vascular imaging, and brain MRI findings. The supportive imaging features included the following:

1. non-lacunar cortical or corticosubcortical infarction, typically presenting as wedge-shaped or irregular lesions;
2. multiple acute infarcts with high signal intensity on Diffusion weighted imaging (DWI), particularly when distributed in different cerebral vascular territories;
3. involvement of multiple cerebral vascular territories, including simultaneous or sequential infarctions in the anterior and posterior circulations.

Patients with alternative evident stroke mechanisms, such as large artery atherosclerosis or small vessel occlusion, were excluded (18).

This retrospective study was conducted in accordance with the Declaration of Helsinki. Written informed consent was obtained from all participants, and the study was reviewed and approved by the Ethics Committee (IRB: 2025-R075).

### 2.2 Clinical data collection

Clinical data of patients were obtained by consulting medical records. Cardiovascular risk factors were recorded, including age, gender, body mass index (BMI), hypertension, hyperlipidemia, diabetes, neutrophil-to-lymphocyte ratio (NLR), N-terminal pro-B-type natriuretic peptide (NT-proBNP), and CHA_2_DS_2_-VASc score. The CHA_2_DS_2_-VASc score (19) is a sum of clinical prediction scores for stroke risk, calculated as follows: 1 point for congestive heart failure (or left ventricular systolic dysfunction); 1 point for hypertension; 2 points for age ≥75 years; 1 point for diabetes; 2 points for previous stroke, transient ischemic attack, or thromboembolism; 1 point for vascular disease; 1 point for 65–74 years; and 1 point for female gender.

### 2.3 CT image analysis

All CT examinations were performed using a 256-slice spiral CT scanner (Philips Healthcare, Cleveland, OH, USA). Patients were positioned supine, with retrospective electrocardiographic gating and respiratory control. Non-ionic iohexanol (350 mgl/mL, dose 0.8 mL/kg) was administered through injection. The scanning range extended from the tracheal bifurcation to 0.5 cm below the transverse septum. Imaging parameters included the following: tube voltage: 80–120 kV; tube current: 280–350 mAs; collimation: 128 × 0.625; pitch: 0.18; rotation time: 330 ms; matrix: 512 × 512; Field of View (FOV): 250 mm; slice thickness: 0.9 mm; and slice spacing: 0.45 mm.

The original images were reconstructed at the 75% phase and transmitted to the post-processing workstation (Philips EBW 4.5), where cardiac analysis was performed using Comprehensive Cardiac Analysis (CCA) software. Initially, myocardial thickness at the LA–LAA junction was measured on three consecutive cross-sectional images (Figures 1A–C). The thickest layer of the myocardium was identified at the isthmus of the LAA (Figure 1A) (20). When the marker was positioned on the image with the thickest myocardium, it was automatically placed at the corresponding position in the sagittal plane image (Figure 1D). Finally, its position was verified on the sagittal image to confirm the thickest layer of the myocardium.

**Figure 1:**
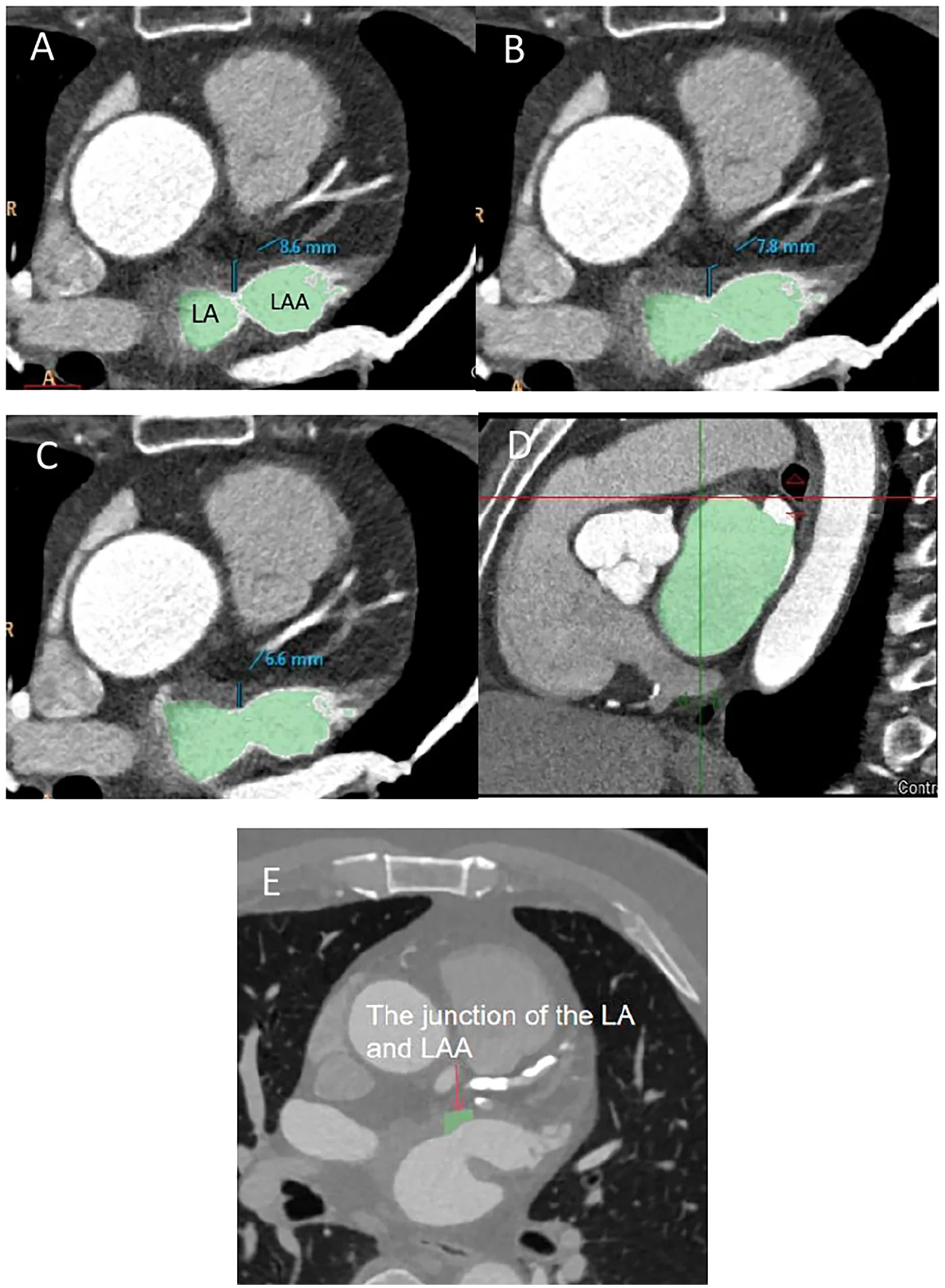
Measurement of myocardial thickness at the LA–LAA junction. **(A–C)** Three consecutive cross-sectional images of the myocardium at the LA–LAA junction. The thickness of three layers of myocardium was measured, with values of 8.6, 7.8, and 6.6 mm. Panel **(A)** has been identified as the thickest myocardial layer. **(D)** The thickest myocardial layer in the cross-sectional image corresponds to the sagittal image. Its position is determined by locating markers on the myocardium of the aforementioned cross-section. **(E)** ROI of the myocardium at the LA–LAA junction delineated on an axial image of cardiac CT (green). The length of ROI was 1 cm in total. LA, left atrial; LAA, left atrium appendage.

### 2.4 Radiomics feature extraction

In this study, ROIs were delineated using 3D Slicer software in the AF-related cardioembolic stroke group and the non-AF-non-stroke group. ROIs were drawn on six optimal layers of the myocardium at the LA–LAA junction. The center of each ROI was assigned as the junction point of the LA and LAA. Ultimately, the ROI was extended 0.5 cm on both sides along the LA and LAA, and the myocardium within this specified range was outlined (Figure 1E). Two experienced radiologists sketched independently to avoid bias, and if there was disagreement about the ROI, consensus was reached in consultation with a third radiologist. The Pyradiomics package in Python software was used to extract radiomics features and generate datasets. A total of 128 radiomics features were extracted, including first-order features, shape features, and texture features (21).

### 2.5 Radiomics dimensionality reduction and model establishment

The Pyradiomics toolkit was used for feature screening and model construction. First, the extracted radiomics feature data were normalized and randomly divided into a training set and a testing set at a ratio of 7:3. The training set data were subjected to dimensionality reduction analysis through T-test and Least Absolute Shrinkage and Selection Operator (LASSO) regression to select the radiomics features with the highest predictive ability. LASSO can compress the weights of unimportant variables to zero to automatically select radiomics features that have a significant impact on the target variable (due to regularization penalties, LASSO tends to reduce weight to zero) (22, 23). Next, we constructed a radiomics model to predict the risk of stroke in AF using support vector machine (SVM) classifiers. We performed 10-fold cross-validation on the training set to identify the most suitable parameters and enhance the stability of the model.

The radiomics score (Rad-score) was calculated by multiplying the screened feature values by their corresponding non-zero weights and summing the products.

$$\mathrm{Rad}\text{-}\mathrm{score}=\beta_{0}+\beta_{1}X_{1}+\beta_{2}X_{2}+\cdots +\beta_{n}X_{n}$$

### 2.6 The clinical characteristics model

Variables with *p* < 0.05 in univariate analysis were selected to construct a clinical model for predicting the risk of AF-related cardioembolic stroke using R Studio software.

### 2.7 The predictive model combining radiomics and clinical characteristics

Variables with *p* < 0.05 in univariate analysis were selected. A model combining the radiomics and the clinical model was constructed to predict the risk of cardioembolic stroke in AF, and a stroke prediction score was calculated.

### 2.8 Nomogram establishment

A nomogram is derived from a multivariate logistic regression equation and presented as a visual graph. The corresponding variable scales are established based on the weights of the regression coefficients for all independent variables. Points represent the scores corresponding to each variable. All known variables are connected through points across the axis to obtain the score values corresponding to each independent variable. In this study, radiomics features and clinical features were combined to construct a nomogram that shows the range of values of different variables and their contribution to the predicted risk.

### 2.9 Statistical analysis

Statistical analyses were performed using SPSS software (version 21.0). Continuous data are presented as mean ± standard deviation for normality and median (interquartile range) for non-normality. Categorical data are presented as frequencies (percentages). Normality was assessed using the Shapiro–Wilk test (≤50) for small samples and the Kolmogorov–Smirnov test (>50) for large samples. The independent two-sample t-test was used for continuous data that conformed to normality and variance homogeneity, and the Mann–Whitney U rank-sum test was used for measurement data that did not conform to normality or variance heterogeneity. Categorical data were analyzed using the chi-square test. For multivariable analysis, variables showing statistically significant differences in univariable analyses and/or considered clinically relevant were considered for multivariable logistic regression to identify factors independently associated with AF-related cardioembolic stroke. A nomogram was constructed based on the final multivariable logistic regression model to estimate the individualized probability of AF-related cardioembolic stroke. Model discrimination was evaluated using receiver operating characteristic (ROC) curves and the area under curve (AUC).

Because this was a retrospective exploratory study, no formal sample size calculation was performed before data collection. The sample size was determined by the number of eligible patients available during the study period. The AF-non-stroke group consisted of only 29 patients. Given the relatively small sample size and the high dimensionality of radiomics data, inclusion of this group in radiomics modeling was considered to increase the risk of model instability and overfitting. Therefore, radiomics analysis was restricted to the AF-related cardioembolic stroke group (*n* = 60) and the non-AF-non-stroke reference group (*n* = 53), which had relatively larger and more comparable sample sizes. This radiomics analysis was considered exploratory and was intended to identify imaging features associated with the AF-related cardioembolic stroke phenotype.

As an additional assessment of the precision of the observed effect, the mean difference in Rad-score between the two groups was 4.44 (95% CI, 2.63–6.37), with the confidence interval excluding zero.

## 3 Results

### 3.1 Study population

Among 216 patients who underwent cardiac CT examinations, 98 patients were in the AF group and 118 were in the non-AF group. Due to reasons such as image quality assessment and contraindications, 9 patients of the AF group and 12 of the non-AF group were excluded. Ultimately, 89 cases were included in the AF group and 106 cases were included in the non-AF group (Figure 2).

**Figure 2:**
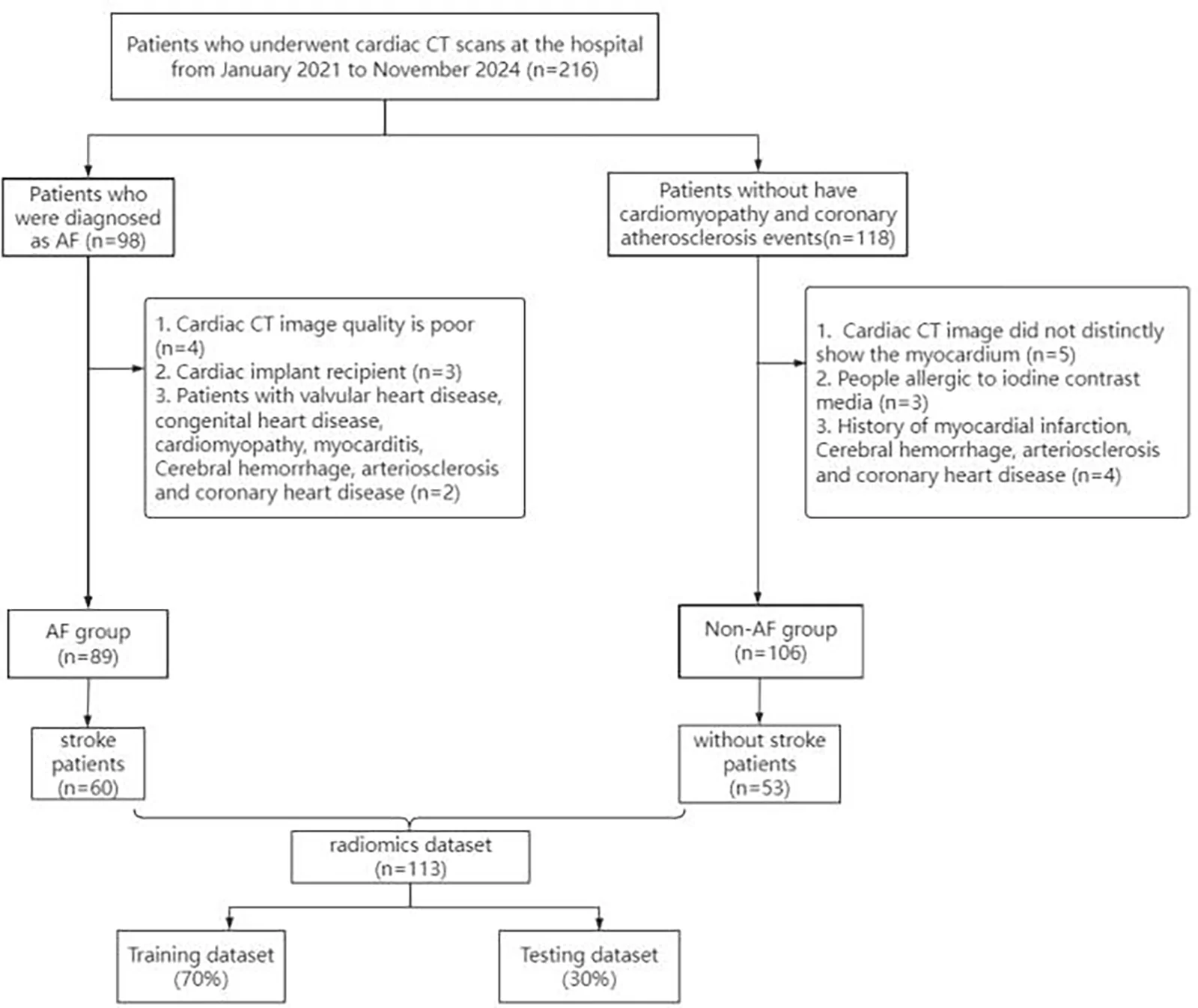
Flowchart of patient recruitment.

Baseline: (1) AF-related cardioembolic stroke (60 cases) vs. AF-non-stroke (29 cases) group; (2) AF-related cardioembolic stroke vs. non-AF-stroke (53 cases) group; and (3) AF-related cardioembolic stroke vs. non-AF-non-stroke (53 cases) group.

### 3.2 The AF-related cardioembolic stroke group vs. AF-non-stroke group

It was found that myocardial thickness at the LA–LAA junction (*p* = 0.049), age (*p* = 0.046), gender (*p* = 0.013), and CHA_2_DS_2_-VASc score (*p* < 0.001) differed significantly between the two groups. Compared with the AF-non-stroke group, patients in the AF-related cardioembolic stroke group had greater myocardial thickness (Table 1).

**Table 1:** Data comparison between the AF-related cardioembolic stroke group and the AF-non-stroke group.

Variable	Univariate			Multivariable		
	AF-related cardioembolic stroke group	AF-non-stroke group	*p*	B	OR	*p*
Age	62.0 (57.0–66.0)	57.60 ± 7.42	0.046*	−0.013	0.987	0.782
Male, *n* (%)	46.0 (85.2%)	15.0 (60.0%)	0.013*	1.748	5.745	0.012*
BMI	26.50 ± 3.59	26.33 ± 2.01	0.817			
Hypertension, *n* (%)	29.0 (53.7%)	8.0 (32.0%)	0.072			
Hyperlipidemia, *n* (%)	12.0 (22.2%)	2.0 (8.0%)	0.124			
Diabetes, *n* (%)	4.0 (7.4%)	4.0 (16.0%)	0.239			
CHA_2_DS_2_-VASc score	3.0 (2.00–4.00)	1.0 (0.50–2.00)	<0.001*	0.960	2.612	<0.001*
NLR	1.8 (1.32–2.40)	1.6 (0.97–2.71)	0.390			
NT-proBNP	265.0 (126.8–1,105.0)	186.6 (63.0–485.0)	0.126			
AF onset times	104.0 (20.3, 409.5)	50.0 (12.0, 668.0)	0.396			
Myocardial thickness	7.1 (6.43,8.33)	6.58 ± 1.45	0.049*	0.245	1.278	0.254

AF, atrial fibrillation; BMI, body mass index; NLR, neutrophil-to-lymphocyte ratio; NT-proBNP, N-terminal pro-B-type natriuretic peptide.

* *p* < 0.05.

Multivariate logistic regression analysis included age, gender, CHA_2_DS_2_-VASc score, and myocardial thickness at the LA–LAA junction. The results showed that gender and CHA_2_DS_2_-VASc score were statistically significant predictors of stroke (Table 1). For stroke patients with a history of AF, each 1-point increase in the CHA_2_DS_2_-VASc score was associated with a 2.612-fold increased risk of AF-related cardioembolic stroke.

### 3.3 The AF-related cardioembolic stroke group vs. non-AF-stroke group

The myocardial thickness at the LA–LAA junction (*p* < 0.001) and NT-proBNP levels (*p* < 0.001) differed significantly between the two groups. In AF patients with a history of stroke, multivariate logistic regression analysis found that only gender was a statistically significant predictor of AF (Table 2).

**Table 2:** Data comparison between the AF-related cardioembolic stroke group and the non-AF-stroke group.

Variable	Univariate			Multivariable		
	AF-related cardioembolic stroke group	Non-AF-stroke group	*p*	B	OR	*p*
Age	62.5 (57.0–66.0)	60.0 (56.5–63.0)	0.077			
Male, *n* (%)	50.0 (83.3%)	19.0 (35.8%)	<0.001*	2.279	9.766	0.002*
BMI	26.44 ± 3.60	25.46 ± 3.65	0.152			
Hypertension, *n* (%)	32.0 (53.3%)	28.0 (52.8%)	0.957			
Hyperlipidemia, n (%)	14.0 (23.3%)	12.0 (22.6%)	0.931			
Diabetes, *n* (%)	6.0 (10.0%)	11.0 (20.8%)	0.111			
CHA_2_DS_2_-VASc score	3.5 (2.00–4.00)	3.0 (1.50–4.00)	0.037*	0.453	1.180	0.453
NLR	1.8 (1.32–2.48)	2.1 (1.29–2.88)	0.216			
NT-proBNP	263.0 (114.5–1,005.0)	66.0 (28.0–144.0)	<0.001*	0.001	1.001	0.272
AF onset times	99.5 (21.5–346.5)	—	—			
Myocardial thickness	7.07 ± 1.46	5.42 ± 1.42	<0.001*	0.105	1.562	0.105

AF, atrial fibrillation; BMI, body mass index; NLR, neutrophil-to-lymphocyte ratio; NT-proBNP, N-terminal pro-B-type natriuretic peptide.

* *p* < 0.05.

In addition, correlation analysis was conducted between myocardial thickness and gender, CHA_2_DS_2_-VASc score, and NT-proBNP. Myocardial thickness was correlated with male gender and NT-proBNP, but not with the CHA_2_DS_2_-VASc score (Supplementary Table S1).

### 3.4 The AF-related cardioembolic stroke group versus non-AF-non-stroke group

There were statistically significant differences in gender (*p* = 0.002), age (*p* < 0.001), CHA_2_DS_2_-VASc score (*p* < 0.001), the myocardial thickness at the LA–LAA junction (*p* < 0.001), and NT-proBNP (*p* < 0.001) between the two groups (Table 3).

**Table 3:** Data comparison between the AF-related cardioembolic stroke group and the non-AF-non-stroke group.

	Univariable			Multivariable		
Variable	AF-related cardioembolic stroke group	Non-AF-non-stroke group	*p*	B	OR	*p*
Age (years)	62.5 (57.0–66.0)	48.15 ± 10.61	<0.001*	0.001	1.000	0.998
Male, *n* (%)	41.0 (80.4%)	27.0 (50.9%)	0.002*	1.838	6.281	0.181
BMI	26.44 ± 3.60	25.8 (24.1–29.0)	0.883			
Hypertension, *n* (%)	28.0 (54.9%)	24.0 (45.3%)	0.327			
Hyperlipidemia, *n* (%)	10.0 (19.6%)	18.0 (34.0%)	0.099			
Diabetes, *n* (%)	5.0 (9.8%)	0	0.060			
CHA_2_DS_2_-VASc score	3.5 (2.00–4.00)	1.0 (1.00–2.00)	<0.001*	1.361	3.899	0.058
NLR	1.82 (1.31–2.48)	2.14 (1.53–2.74)	0.100			
NT-proBNP (pg/mL)	263.0 (114.5–1,005.0)	24.5 (15.0–63.13)	<0.001*	0.011	1.011	0.110
Myocardial thickness (mm)	7.07 ± 1.46	5.14 ± 1.02	<0.001*	2.086	8.051	0.048*

AF, atrial fibrillation; BMI, body mass index; NLR, neutrophil-to-lymphocyte ratio; NT-proBNP, N-terminal pro-B-type natriuretic peptide.

* *p* < 0.05.

The results of multivariate logistic regression analysis showed that myocardial thickness (*p* = 0.048) significantly impacted AF-related cardioembolic stroke.

### 3.5 Radiomics analysis between AF-related cardioembolic stroke group and non-AF-non-stroke group

Three features with the best predictive effects were selected from the 128 extracted radiomics features (Figure 3A). In LASSO regression, the adjustment parameter *λ* was used. When *λ* reached its optimal value, LASSO showed the best predictive ability, screening out the important features (Figures 3B,C). Subsequently, a radiomics model was constructed.

**Figure 3:**
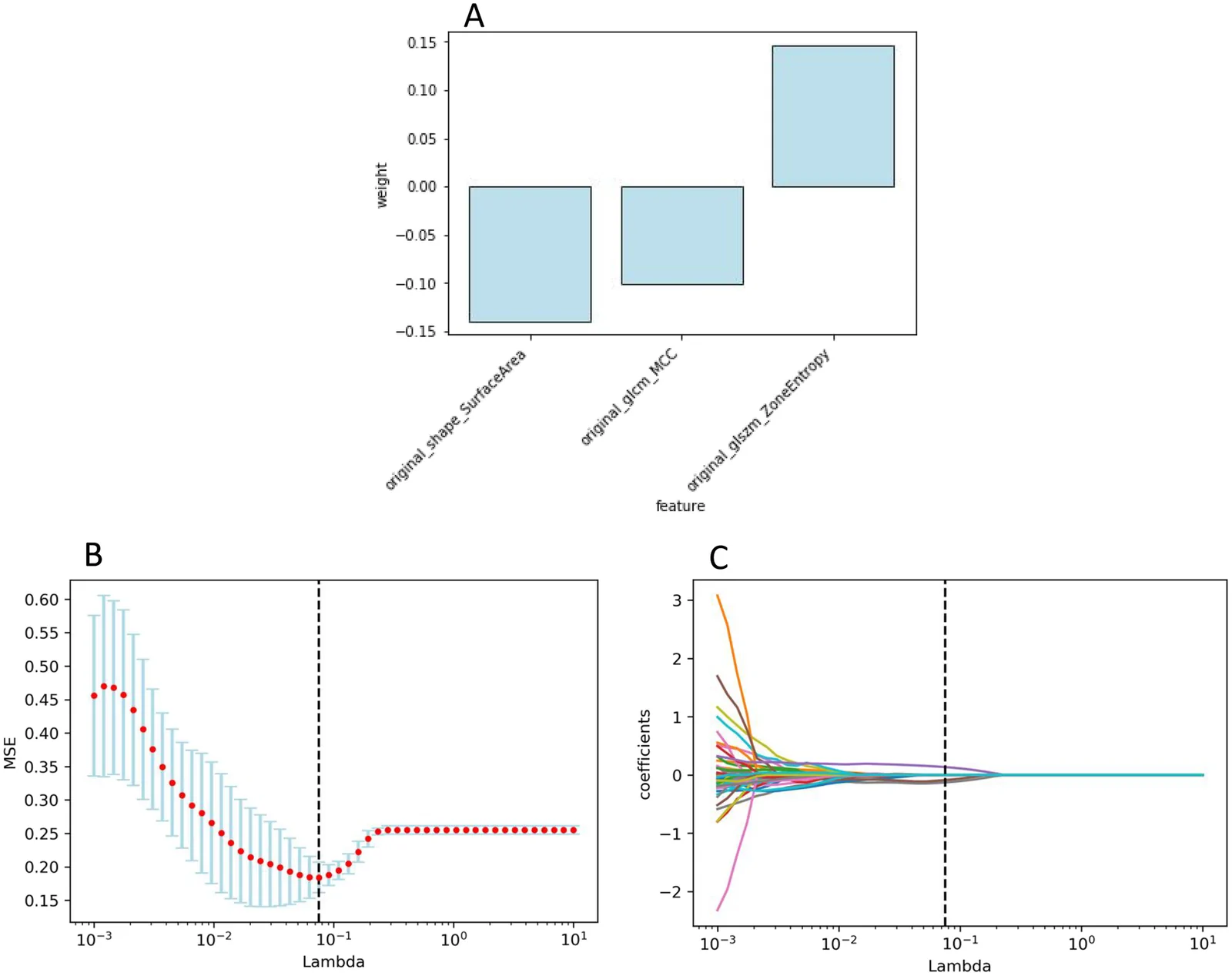
**(A)** The radiomics features and weight values screened out. Three features are screened out. The names of these features are displayed on the *X*-axis, and the corresponding feature weight values are shown on the *Y*-axis. Positive or negative weights indicate a positive or negative correlation with AF-related cardioembolic stroke. **(B)** Dimensionality reduction with LAASO regression model. *λ* in the shrinkage coefficient represents the adjustment parameter. The vertical axis represents the mean square error, and the horizontal axis represents *λ*. **(C)** Feature coefficients with *λ* change. As the *λ* increases, the coefficients are gradually compressed to 0, thereby screening out the important features, making the model more stable.

A Rad-score calculation formula was derived based on the selected features. According to this formula, the Rad-score for each patient can ultimately be calculated. The formula was as follows:

Rad-score=(−0.139384×feature1)+(−0.101318×feature2)+(0.146422×feature3)

The results showed that the Rad-score of the AF-related cardioembolic stroke group was lower than that of the non-stroke group (*p* < 0.001) (Supplementary Table S2).

### 3.6 Prediction model of AF-related cardioembolic stroke

The present study constructed three predictive models to assess the risk of AF-related cardioembolic stroke, including a model based on clinical features, a model based on radiomics features, and a combined model integrating clinical and radiomics features.

#### 3.6.1 Clinical model

In univariate analysis, it was found that age, gender, CHA_2_DS_2_-VASc score, and the myocardial thickness at the LA–LAA junction were associated with the occurrence of AF-related cardioembolic stroke. A clinical multivariate logistic regression model was established by incorporating these variables.

#### 3.6.2 Nomogram construction based on the radiomics combined with clinical feature model

A predictive model was established by combining clinical features and Rad-score. The model was visualized as a nomogram (Figure 4A). Calibration curves demonstrated good consistency between the model's predictions and actual outcomes (Figure 4B).

**Figure 4:**
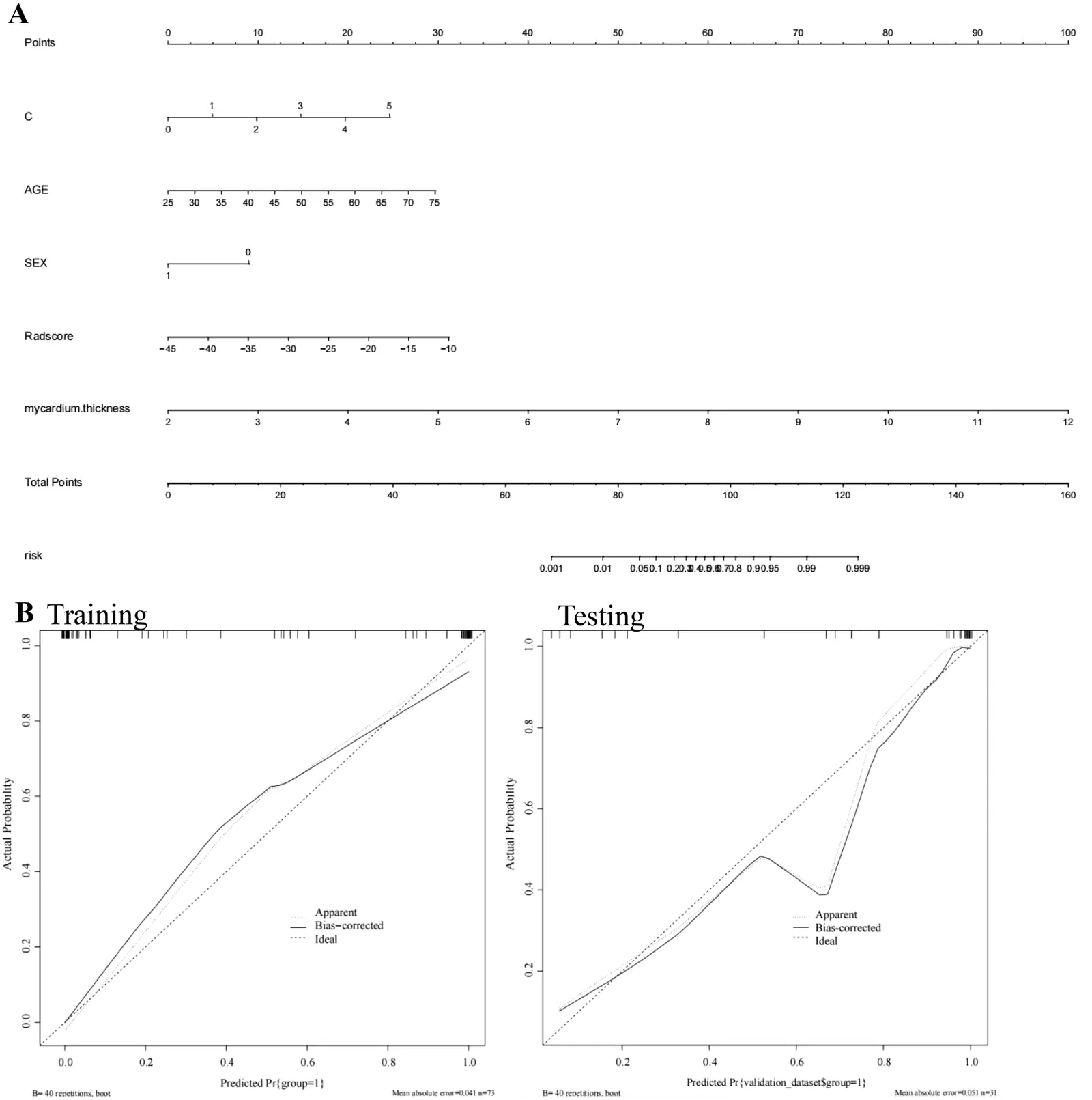
**(A)** A nomogram combining radiomics and clinical features model. **(B)** Calibration curves of the combination of radiomics and clinical features model. When the solid line (predicted situation) is closer to the dashed line (actual situation), the calibration effect of the model is better.

### 3.7 Model performance evaluation

The model combining radiomics and clinical features had an AUC of 0.968, which was higher than that of the radiomics model (0.878) and the clinical features model (0.955) (Figure 5).

**Figure 5:**
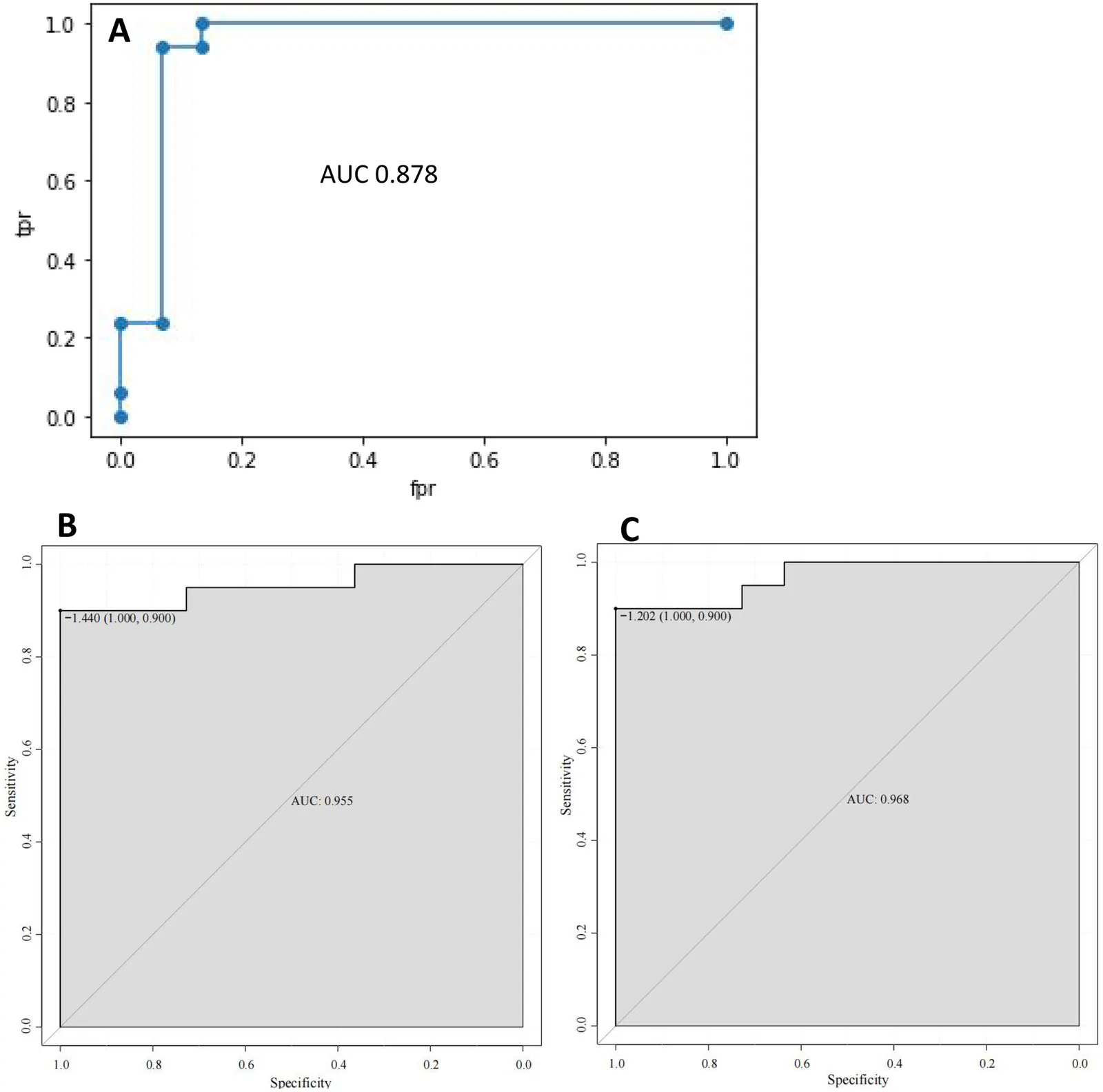
ROC curves. **(A)** ROC curve of radiomics model; **(B)** ROC curve of clinical feature model; **(C)** ROC curve of the combined radiomics and clinical features model.

Furthermore, age, gender, CHA_2_DS_2_-VASc score, Rad-score, and myocardial thickness were used to establish an equation for predicting the probability of stroke occurrence:

$$\begin{array}{rl} P= & 1/(1+\exp\;(-(\beta_{1}\times \mathrm{Age}+\beta_{2}\times gender+\beta_{3}\\ & \times \mathrm{CH}A_{2}DS_{2}\text{-}\mathrm{VASc}\;\mathrm{score}+\beta_{4}\times \mathrm{Rad}\text{-}\mathrm{score}\\ & +\beta_{5}\times \mathrm{Myocardial}\;\mathrm{thickness}))) \end{array}$$

### 3.8 Correlation between myocardial thickness and LAA emptying function

There were significant differences in myocardial thickness (*p* = 0.011), CHA_2_DS_2_-VASc score (*p* = 0.001), NT-proBNP levels (*p* < 0.001), and the prevalence of hypertension (*p* = 0.025) between AF patients with reduced and normal LAA emptying function. Patients with reduced LAA emptying function exhibited greater myocardial thickness, higher CHA_2_DS_2_-VASc score and NT-proBNP levels, and a higher prevalence of hypertension (Table 4). Myocardial thickness, CHA_2_DS_2_-VASc score, NT-proBNP levels, and hypertension were all significantly associated with LAA emptying function. LAA emptying function demonstrated a strong negative correlation with NT-proBNP.

**Table 4:** Comparing myocardial thickness with different LAA emptying functions in the AF group.

Variable	Reduced LAA emptying function	Normal LAA emptying function	Correlation Coefficient	*p*
Age (years)	60.45 ± 7.26	60.0 (54.25–65.0)		0.418
Male, n(%)	19.0 (86.3%)	28.0 (70.0%)		0.150
BMI	26.19 (25.11–29.4)	26.03 ± 3.07		0.160
Hypertension, *n* (%)*	13.0 (59.0%)	12.0 (30.0%)	−2.84	0.025*
Hyperlipidemia, *n* (%)	4.0 (18.1%)	9.0 (22.5%)		0.689
Diabetes, *n* (%)	2.0 (9.0%)	6.0 (15.0%)		0.507
CHA_2_DS_2_-VASc score*	3.64 ± 1.36	2.0 (1.00–3.75)	−4.08	0.001*
NLR	1.92 ± 0.78	1.30 (0.96–2.15)		0.130
NT-proBNP (pg/mL)*	695.5 (348.25–1,338.65)	117.0 (51.0–262.5)	−6.34	<0.001*
AF onset times	90.0 (12.75–473.25)	87.0 (20.5–392.75)		0.654
Myocardial thickness (mm)	7.44 ± 1.06	6.56 ± 1.35	−3.46	0.011*

AF, atrial fibrillation; BMI, body mass index; NLR, neutrophil-to-lymphocyte ratio; NT-proBNP, N-terminal pro-B-type natriuretic peptide.

* *p* < 0.05.

## 4 Discussion

This study systematically evaluated the changes in myocardium at the LA–LAA junction in patients with AF-related cardioembolic ischemic stroke. The results showed that radiomics features and thickness of myocardium at the LA–LAA junction significantly changed in patients with AF-related cardioembolic stroke. These parameters may serve as potential imaging indicators for differentiating the AF-related cardioembolic stroke status from the healthy state non-AF-non-stroke. Moreover, the model combining radiomics features with clinical parameters demonstrated good discrimination ability in identifying AF-related cardioembolic stroke (AUC = 0.968), suggesting that the radiomics features of the myocardium in this region can serve as a potential imaging marker for stroke risk stratification in AF patients.

Previous studies have shown that AF increases the risk of stroke by approximately fivefold (24). This is consistent with our observation that AF patients are more prone to stroke. In addition, the male predominance observed in the AF-related cardioembolic stroke group largely reflects the higher proportion of male participants in our overall cohort. Moreover, previous studies have found that the incidence of AF is higher in men than in women (25). In addition, this study found that the CHA_2_DS_2_-VASc score in the AF-related cardioembolic stroke group was higher than that in the AF-non-stroke group. This score is a well-established and effective AF-related cardioembolic stroke risk stratification scale (26). Previous studies have also shown that patients with LAA thrombosis have a higher CHA_2_DS_2_-VASc score (27). The CHA_2_DS_2_-VASc score can effectively assess stroke caused by cardioembolism.

Multivariable logistic regression analysis comparing the AF-related cardioembolic stroke and AF-non-stroke groups showed that, after adjustment for age, gender, and CHA2DS2-VASc score, myocardial thickness at the LA–LAA junction was not independently associated with stroke, whereas the CHA2DS2-VASc score and gender remained significantly associated with stroke. This finding suggests that the association between LA–LAA junction myocardial thickness and cardioembolic stroke may overlap, at least in part, with established clinical risk factors captured by the CHA2DS2-VASc score. The CHA2DS2-VASc (24) score exhibited stronger independent predictive utility in our AF cohort. However, LA–LAA myocardial thickness may represent structural atrial remodeling and may provide complementary imaging information, which warrants further evaluation in larger prospective cohorts.

NT-proBNP levels were significantly higher in the AF-related cardioembolic stroke group compared with the other three groups. NT-proBNP, a cardiac-secreted protein fragment, is a well-established biomarker for cardiac function assessment and cardiovascular disease diagnosis, particularly in heart failure (28). Importantly, it has also been recognized as a marker of AF occurrence (29), with elevated NT-proBNP levels observed during AF episodes—a finding consistent with a report by Sepehri Shamloo et al. (2). Furthermore, NT-proBNP has been linked to cardioembolic stroke (30) and serves as a marker of thromboembolic risk in AF patients (31).

In the comparison between the AF-related cardioembolic stroke group and the non-AF-non-stroke group, myocardial thickness demonstrated independent predictive value, indicating that this metric effectively differentiates between two distinct states and reveals the combined pathological effects of AF and stroke. Through radiomics feature selection, three features were identified with the highest weights in distinguishing these two groups. The first feature, original_shape_SurfaceArea, was a macroscopic geometric index that captures myocardial morphological changes at the LA–LAA junction. The second, original_glcm_MCC, quantified myocardial micro-heterogeneity and distinguished textural differences between remodeled and normal myocardium. The third, original_glszm_ZoneEntropy, was a sensitive indicator of tissue heterogeneity changes, with higher values suggesting more pronounced myocardial fibrosis or microstructural disruption. Collectively, these findings indicate that the myocardium exhibits significant alterations in geometric configuration, textural patterns, and internal homogeneity. In addition, AF-related cardioembolic stroke patients presented with thicker myocardium and significantly lower Rad-scores, underscoring that microstructural myocardial changes are a prominent characteristic in this population (32). ROC analysis demonstrated excellent predictive performance of the combined model (AUC = 0.968), providing strong support for its application in individualized stroke risk stratification. Calibration curve analysis further confirmed good agreement between predicted and observed outcomes, indicating favorable predictive performance.

Hypertension and myocardial thickness were associated with LAA emptying function. The flow of blood from the LAA cavity to the LA cavity is defined as LAA emptying (33). In AF patients, reduced LAA emptying velocity—particularly values below 40 cm/s—significantly increases the likelihood of LAA thrombus formation (34), thereby elevating the risk of cardioembolic stroke (35). This study further revealed that myocardial thickness at the LA–LAA junction was thicker in AF patients with impaired LAA emptying than in those with normal emptying function, suggesting a possible link to AF-related myocardial fibrosis.

Speckle-tracking echocardiography (STE) has also been used to identify LAA thrombus in AF patients (36). Previous studies have demonstrated that STE-derived LA and LAA strain parameters perform well in identifying AF patients with a higher probability of LAA spontaneous echo contrast (SEC) and thrombus formation. Moreover, 4D Strain STE can effectively assess the myocardium (37). In contrast to STE, cardiac CT allows accurate assessment of myocardial thickness and LAA morphology, and is less operator-dependent. Dual-energy CT (DECT) with delayed phase imaging eliminates the influence of slow blood flow in the LAA, enhances contrast between blood flow and thrombus, and significantly improves the diagnostic accuracy for LAA thrombus detection (38). Multiple studies have confirmed that DECT is a reliable tool for evaluating LAA thrombosis (39, 40).

## 5 Limitations

This study had the following limitations. First, radiomics analysis only included two groups: AF-related cardioembolic stroke and non-AF-non-stroke. The AF-non-stroke group was not included in the modeling due to insufficient sample size (*n* = 29), due to which the radiomics differences could not be solely attributed to AF-related thrombus formation. Second, this model is a retrospective discrimination model, distinguishing between individuals who have and have not experienced stroke, rather than serving as a prospective stroke risk prediction tool. It needs external validation in an independent cohort. Third, this study was a single-center retrospective study with a relatively modest sample size. Multicenter, prospective, and large-scale studies are needed for further validation. In spite of these limitations, this study is the first to adopt a case–control design, systematically reflecting the independent and combined effects of AF and stroke on the myocardium at the LA–LAA junction. The results demonstrate that increased myocardial thickness in this region primarily reflects chronic structural remodeling related to AF rather than acute stroke events. The radiomics features of the myocardium in this region hold promise as structural imaging markers for stratifying the risk of AF-related cardioembolic stroke in patients.

## 6 Conclusions

Both myocardial radiomics features and thickness of the myocardium at the LA–LAA junction may serve as potential imaging markers associated with AF-related cardioembolic stroke. Their incremental value for stroke risk stratification beyond established clinical risk factors requires further validation in larger prospective cohorts.

## Data Availability

The raw data supporting the conclusions of this article will be made available by the authors without undue reservation.

## References

[1] Yang M Cao Q Xu Z Ge Y Li S Yan F. Development and validation of a machine learning-based radiomics model on cardiac computed tomography of epicardial adipose tissue in predicting characteristics and recurrence of atrial fibrillation. Front Cardiovasc Med. (2022) 9:813085. 10.3389/fcvm.2022.813085 35310976 PMC8927627

[2] Sepehri Shamloo A Bollmann A Dagres N Hindricks G Arya A. Natriuretic peptides: biomarkers for atrial fibrillation management. Clin Res Cardiol. (2020) 109:957–66. 10.1007/s00392-020-01608-x 32002634

[3] Escudero-Martínez I Ocete RF Mancha F Vega Á Piñero P López-Rueda A. Prevalence and risk factors of silent brain infarcts in patients with AF detected by 3T-MRI. J Neurol. (2020) 267:2675–82. 10.1007/s00415-020-09887-0 32410017

[4] Escudero-Martínez I Morales-Caba L Segura T. Atrial fibrillation and stroke: a review and new insights. Trends Cardiovasc Med. (2021) 33:23–9. 10.1016/j.tcm.2021.12.001 34890796

[5] Chen Y He Y Jiang Z Xie Y Nie S. Ischemic stroke subtyping method combining convolutional neural network and radiomics. J Xray Sci Technol. (2023) 31:223–35. 10.3233/XST-221284 36591693 PMC10041412

[6] Ozdemir H Sagris D Lip GYH Abdul-Rahim AH. Stroke in atrial fibrillation and other atrial dysrhythmias. Curr Cardiol Rep. (2023) 25:357–69. 10.1007/s11886-023-01862-1 36976496

[7] Chahine Y Akoum N. Fibrosis: a nexus between atrial fibrillation and left atrial appendage thrombosis. J Cardiovasc Electrophysiol. (2022) 33:688–9. 10.1111/jce.15382 35064708

[8] Safavi-Naeini P Rasekh A. Thromboembolism in atrial fibrillation: role of the left atrial appendage. Card Electrophysiol Clin. (2020) 12:13–20. 10.1016/j.ccep.2019.11.003 32067643

[9] Tabak C Smith R Bajaj M Baghdadi S Parikh R Enders R. Predictors of left atrial appendage emptying velocity: derivation and validation of CHIRP(3)(M(-1)) score. Heart Rhythm. (2024) 22:923–31. 10.1016/j.hrthm.2024.09.065 39370027 PMC13719310

[10] Pan J Kramer C. Cardiac magnetic resonance imaging in heart failure. Cardiovasc Innov Appl. (2024) 9(1):1–20. 10.15212/CVIA.2024.0037 PMC12380259 40873888

[11] Tang Y Ma X Dong ZX Chen XG Yang SJ Chen XY. Cardiac magnetic resonance visualization of the myocardial microstructure in non-ischemic cardiomyopathies. Cardiovasc Innov Appl. (2024) 9:1–6. 10.15212/CVIA.2024.0042

[12] Lambin P Rios-Velazquez E Leijenaar R Carvalho S Van Stiphout RGPM Granton P. Radiomics: extracting more information from medical images using advanced feature analysis. Eur J Cancer. (2012) 48:441–6. 10.1016/j.ejca.2011.11.036 22257792 PMC4533986

[13] Mariotti F Agostini A Borgheresi A Marchegiani M Zannotti A Giacomelli G. Insights into radiomics: a comprehensive review for beginners. Clin Transl Oncol. (2025) 27:4091–102. 10.1007/s12094-025-03939-5 40355777 PMC12559095

[14] Ismail FA Abdul Karim MK Kechik MMA Kamal I Ismail Z Mohd Salim E. Radiomics-based machine learning models for classifying breast cancer on dynamic-contrast enhanced MRI through multi-observer analysis. Curr Med Imaging. (2026) 22:e15734056403426. 10.2174/0115734056403426251205212737 41820213 PMC13445547

[15] Pujadas ER Raisi-Estabragh Z Szabo L McCracken C Morcillo CI Campello VM. Prediction of incident cardiovascular events using machine learning and CMR radiomics. Eur Radiol. (2022) 33:3488–500. 10.1007/s00330-022-09323-z 36512045 PMC10121487

[16] Feng E Qin P Chai R Zeng J Wang Q Meng Y. MRI generated from CT for acute ischemic stroke combining radiomics and generative adversarial networks. IEEE J Biomed Health Inform. (2022) 26:6047–57. 10.1109/JBHI.2022.3205961 36094997

[17] Pujadas ER Raisi-Estabragh Z Szabo L Morcillo CI Campello VM Martin-Isla C. Atrial fibrillation prediction by combining ECG markers and CMR radiomics. Sci Rep. (2022) 12:18876. 10.1038/s41598-022-21663-w 36344532 PMC9640662

[18] Sreedharan SE Saikiran K Chandralekha SC Athira CA Sylaja PN Jissa VT. Clinical, imaging, and atrial cardiopathy markers in ischemic stroke subtypes—clues to a cardioembolic source. Ann Indian Acad Neurol. (2025) 28:568–73. 10.4103/aian.aian_772_24 39794287 PMC12393849

[19] Hindricks G Potpara T Dagres N Arbelo E Bax JJ Blomström-Lundqvist C. 2020 ESC guidelines for the diagnosis and management of atrial fibrillation developed in collaboration with the European Association for Cardio-Thoracic Surgery (EACTS): the task force for the diagnosis and management of atrial fibrillation of the European Society of Cardiology (ESC) developed with the special contribution of the European Heart Rhythm Association (EHRA) of the ESC. Eur Heart J. (2020) 42:373–498. 10.1093/eurheartj/ehaa612 32860505

[20] Hołda MK Hołda J Strona M Koziej M Klimek-Piotrowska W. Blood vessels and myocardial thickness within the left atrial appendage isthmus line. Clin Anat. (2018) 31:1024–30. 10.1002/ca.23242 30069992

[21] Kolossváry M Kellermayer M Merkely B Maurovich-Horvat P. Cardiac computed tomography radiomics: a comprehensive review on radiomic techniques. J Thorac Imaging. (2017) 33:26–34. 10.1097/RTI.0000000000000268 28346329

[22] Dai P Chang W Xin Z Cheng H Ouyang W Luo A. Retrospective study on the influencing factors and prediction of hospitalization expenses for chronic renal failure in China based on random forest and LASSO regression. Front Public Health. (2021) 9:678276. 10.3389/fpubh.2021.678276 34211956 PMC8239170

[23] Zheng Y-M Chen J Xu Q Zhao W-H Wang X-F Yuan M-M. Development and validation of an MRI-based radiomics nomogram for distinguishing Warthin's tumour from pleomorphic adenomas of the parotid gland. Dentomaxillofac Radiol. (2021) 50:20210023. 10.1259/dmfr.20210023 33950705 PMC8474129

[24] Migdady I Russman A Buletko AB. Atrial fibrillation and ischemic stroke: a clinical review. Semin Neurol. (2021) 41:348–64. 10.1055/s-0041-1726332 33851396

[25] Yang S De Kruijf N Zhu F Van Oortmerssen JAE De Groot NMS Kavousi M. Trends in burden of atrial fibrillation over three decades: a population-based study. Heart. (2025):1–10. 10.1136/heartjnl-2025-326510 41015500

[26] Camm AJ Kirchhof P Lip GYH Schotten U Savelieva I Ernst S. Guidelines for the management of atrial fibrillation: the Task Force for the Management of Atrial Fibrillation of the European Society of Cardiology (ESC). Eur Heart J. (2010) 31:2369–429. 10.1093/eurheartj/ehq278 20802247

[27] Kupczynska K Michalski BW Miskowiec D. Association between left atrial function assessed by speckle-tracking echocardiography and the presence of left atrial appendage thrombus in patients with atrial fibrillation. Anatol J Cardiol. (2017) 18:15–22. 10.14744/AnatolJCardiol.2017.7613 28559531 PMC5512193

[28] Almeida AG. NT-proBNP and myocardial fibrosis: the invisible link between health and disease. J Am Coll Cardiol. (2017) 70:3110–2. 10.1016/j.jacc.2017.11.010 29268925

[29] Dudink EA Weijs B Tull S. The biomarkers NT-proBNP and CA-125 are elevated in patients with idiopathic atrial fibrillation. J Atr Fibrillation. (2019) 11:2058. 10.4022/jafib.2058 PMC6533832 31139280

[30] Di Castelnuovo A Veronesi G Costanzo S Zeller T Schnabel RB De Curtis A. NT-proBNP (N-terminal pro-B-type natriuretic peptide) and the risk of stroke. Stroke. (2019) 50:610–7. 10.1161/STROKEAHA.118.023218 30786848

[31] Shimizu H Murakami Y Inoue S-i Ohta Y Nakamura K Katoh H. High plasma brain natriuretic polypeptide level as a marker of risk for thromboembolism in patients with nonvalvular atrial fibrillation. Stroke. (2002) 33:1005–10. 10.1161/hs0402.105657 11935052

[32] Watson CJ Glezeva N Horgan S Gallagher J Phelan D McDonald K. Atrial tissue pro-fibrotic M2 macrophage marker CD163+, gene expression of procollagen and B-type natriuretic peptide. J Am Heart Assoc. (2020) 9:e013416. 10.1161/JAHA.119.013416 32431194 PMC7428985

[33] Hwang SH Oh Y-W Kim M-N Park S-M Shim WJ Shim J. Relationship between left atrial appendage emptying and left atrial function using cardiac magnetic resonance in patients with atrial fibrillation: comparison with transesophageal echocardiography. Int J Cardiovasc Imaging. (2016) 32:163–71. 10.1007/s10554-016-0893-1 27119163

[34] Xu X Liao W Huang Q Chen J Bu M Fu Z. FGF-23: a novel and critical predictor of reduced left atrial appendage emptying velocity in patients with atrial fibrillation. Cardiovasc Innov Appl. (2025) 10(1):1–5. 10.15212/CVIA.2025.0007

[35] Beinart R Heist E Newell J Holmvang G Ruskin J Mansour M. Left atrial appendage dimensions predict the risk of stroke/TIA in patients with atrial fibrillation. J Cardiovasc Electrophysiol. (2010) 22:10–5. 10.1111/j.1540-8167.2010.01854.x 20662984

[36] Sonaglioni A Lombardo M Nicolosi GL Gensini GF Ambrosio G. Mechanical concordance between left atrium and left atrial appendage in nonvalvular atrial fibrillation: can it be exploited to avoid transesophageal echocardiography prior to electrical cardioversion during COVID-19 pandemic? Int J Cardiovasc Imaging. (2021) 38:351–62. 10.1007/s10554-021-02414-w 34537932 PMC8449702

[37] Mehrotra A Mehrotra A Shaban M Kacker S. Four-dimensional XStrain echocardiographic assessment of left ventricular strain and rotational mechanics: technology, clinical applications, advantages and limitations. Cardiovasc Innov Appl. (2024) 9(1):1–17. 10.15212/CVIA.2024.0001

[38] Osawa K Nakanishi R Ceponiene I Nezarat N French WJ Budoff MJ. Predicting left atrial appendage thrombus from left atrial volume and confirmation by computed tomography with delayed enhancement. Tex Heart Inst J. (2020) 47(2):78–85. 10.14503/THIJ-17-6290 32603460 PMC7328069

[39] Thomas SG Jacqueline S. Periprocedural imaging for left atrial appendage closure: computed tomography, transesophageal echocardiography, and intracardiac echocardiography. Card Electrophysiol Clin. (2020) 12(1):55–65. 10.1016/j.ccep.2019.11.007 32067648

[40] Li X-N Wang J-X Wei Q Yu X-B Zhou Y-T Ma X-Y. Diagnostic value of delayed contrast-enhanced cardiac computed tomography for detecting left atrial appendage thrombus in patients with atrial fibrillation. Front Cardiovasc Med. (2022) 9(0):847163. 10.3389/fcvm.2022.847163 35571218 PMC9095922

