# Supplementary material for: Correlation between myocardial radiomics features at the left atrium–left atrial appendage junction and stroke risk in atrial fibrillation patients: a retrospective case–control study
Source: Front Cardiovasc Med. 2026 Sep 23;13:1817530. doi: 10.3389/fcvm.2026.1817530 (PMC13641991; doi:10.3389/fcvm.2026.1817530)
Supplement: Supplementary file 1 [file Table1.docx]

**Supplement Table 1 Correlation between male, CHA₂DS₂-VASc score, NT-pro BNP and myocardial thickness at the LA-LAA junctipn**

| variable | Myocardial thickness | *p* |
| --- | --- | --- |
| Male | -0.407 | 0.001 |
| CHA₂DS₂-VASc score | 0.173 | 0.068 |
| NT-pro BNP | 0.381 | 0.002 |

*p*<0.05

AF=atrail fibrillation; NT-pro BNP=N-terminal pro-B-type natriuretic peptide.
