# Supplementary material for: Correlation between myocardial radiomics features at the left atrium–left atrial appendage junction and stroke risk in atrial fibrillation patients: a retrospective case–control study
Source: Front Cardiovasc Med. 2026 Sep 23;13:1817530. doi: 10.3389/fcvm.2026.1817530 (PMC13641991; doi:10.3389/fcvm.2026.1817530)
Supplement: Supplementary file 2 [file Table2.docx]

**Supplement Table 2 Rad-score comparison between the AF-related cardioembolic stroke group and the nonAF-nonstroke group**

| variable | AF-related cardioembolic stroke group | nonAF-nonstroke group | *p* |
| --- | --- | --- | --- |
| Rad-score | -26.27±4.17 | -21.1(-23.30, -18.44) | <0.001 |
